# The functional role of the medial motion area V6

**DOI:** 10.3389/fnbeh.2012.00091

**Published:** 2013-01-16

**Authors:** Sabrina Pitzalis, Patrizia Fattori, Claudio Galletti

**Affiliations:** ^1^Department of Education in Sport and Human Movement, University of Rome “Foro Italico”Rome, Italy; ^2^Laboratory of Neuropsychology, Santa Lucia FoundationRome, Italy; ^3^Department of Human and General Physiology, University of BolognaBologna, Italy

**Keywords:** optic flow, parieto-occipital cortex, dorsal visual stream, MT/V5, wide-field retinotopic mapping, fMRI, visual topography, cortical flattening

## Abstract

In macaque, several visual areas are devoted to analyze motion in the visual field, and V6 is one of these areas. In macaque, area V6 occupies the ventral part of the anterior bank of the parieto-occipital sulcus (POs), is retinotopically-organized and contains a point-to-point representation of the retinal surface. V6 is a motion sensitive area that largely represents the peripheral part of the visual field and whose cells are very sensitive to translational motion. Based on the fact that macaque V6 contains many real-motion cells, it has been suggested that V6 is involved in object-motion recognition. Recently, area V6 has been recognized also in the human brain by neuroimaging and electrophysiological methods. Like macaque V6, human V6 is located in the POs, is retinotopically organized, and represents the entire contralateral hemifield up to the far periphery. Human V6, like macaque V6, is a motion area that responds to unidirectional motion. It has a strong preference for coherent motion and a recent combined VEPs/fMRI work has shown that area V6 is even one of the most early stations coding the motion coherence. Human V6 is highly sensitive to flow field and is also able to distinguish between different 3D flow fields being selective to translational egomotion. This suggests that this area processes visual egomotion signals to extract information about the relative distance of objects, likely in order to act on them, or to avoid them. The view that V6 is involved in the estimation of egomotion has been tested also in other recent fMRI studies. Thus, taken together, human and macaque data suggest that V6 is involved in both object and self-motion recognition. Specifically, V6 could be involved in “subtracting out” self-motion signals across the whole visual field and in providing information about moving objects, particularly during self-motion in a complex and dynamically unstable environment.

## Introduction

Analysis of visual motion has a crucial biological significance, in that it allows an animal or a human being to predict the visual trajectory of moving objects so to allow their grasping or avoid potentially dangerous contact with approaching entities. For a successful action planning, the visuomotor system must recognize if a movement signaled at retinal level is due to an object displacement in the environment or to a self-movement. Consistent with the evolutionary importance of movement detection for safety, several brain regions in the primate dorsal visual pathway are specialized for different aspects of visual motion processing.

The dorsal visual stream begins in the striate cortex (V1), extends through several extrastriate areas, and terminates in higher areas of the parietal and temporal lobes. The middle temporal area (MT or V5) and the middle superior temporal area (MST) are classically considered the key motion regions of the dorsal visual stream, being strongly responsive to visual stimuli in motion and showing selectivity for the direction (e.g., Felleman and Kaas, 1984; Petersen et al., 1985; Newsome et al., 1986; Tanaka et al., 1986; Tootell et al., 1995; Morrone et al., 2000; Smith et al., 2006) and speed (e.g., Allman et al., 1985; Rodman and Albright, 1987; Treue and Andersen, 1996; McKeefry et al., 2008; Lebranchu et al., 2010; Pitzalis et al., 2012a) of movement.

More recently, our group have revealed the presence of another key motion region in the dorsal visual stream, area V6, located medially in the parieto-occipital sulcus (POs) (Galletti et al., 1996, 1999a; Pitzalis et al., 2006, 2010). The functional organization of the macaque area V6 has been originally described on the basis of single cell activity (Galletti et al., 1996, 1999a,b) while that of human V6 was described using cortical-surface-based fMRI mapping techniques, wide-field retinotopic stimulation and electrophysiological methods. Here we review converging evidence that V6 is a retinotopically-organized extrastriate visual area involved in both object and self-motion recognition, providing information about moving objects, particularly during self-motion in a complex and dynamically unstable visual environment.

In the following sections, we will first report a separate description of the main results achieved on area V6 in both macaque and human brain. Then, we will combine the evidence from macaque and human brain to suggest the possible functional role played by area V6, and discuss the functional differences of the medial motion area V6 with respect to the classic lateral motion areas V5/MT and MST.

## Area V6 in the macaque brain

**Figure 1:**
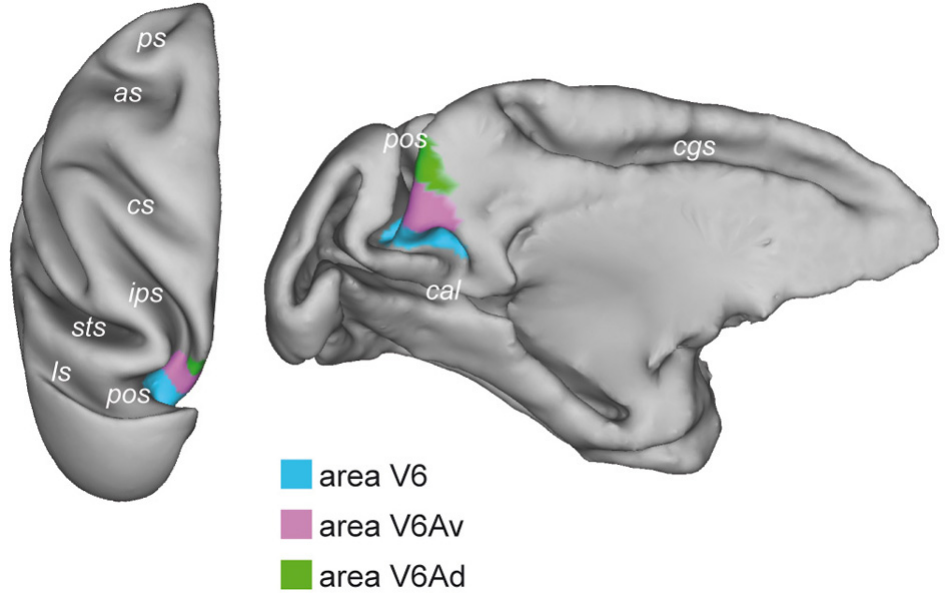
**Brain location of macaque area V6.** Dorsal (**left**) and medial (**right**) views of the surface-based 3D reconstructions of the ATLAS brain of the macaque obtained by CARET (Computerized Anatomical Reconstruction and Editing Toolkit, http://brainvis.wustl.edu/wiki/index.php/Caret:About) (Van Essen et al., 2001) showing the extent of area V6 (light blue) on the left hemisphere. pos, parieto-occipital sulcus; cal, calcarine sulcus; cgs, cingulate sulcus; ips, intraparietal sulcus; sts, superior temporal sulcus; ls, lunate sulcus; cs, central sulcus; as, arcuate sulcus; ps, principal sulcus.

The existence of visual responses in the anterior bank of the POs of the monkey dates back in the 1980s when a visual region was found in anaesthetized animals (Gattass et al., 1985; Colby et al., 1988). This brain region was given the name PO because of its parieto-occipital location. Area PO was described as a strongly myelinated visual region occupying the ventral half of the anterior wall of POs, as well as the ventro-caudal precuneate cortex on the mesial surface of the hemisphere. In awake macaque monkeys, two areas with visual properties have been identified within and nearby the PO territory: area V6 and area V6A (Galletti et al., 1996, 1999a,b, 2005). As shown in Figure 1, the ventral part of the anterior bank of POs is occupied by the visual area V6 (Galletti et al., 1999a), while the dorsal part of the anterior bank of POs is occupied by the visuomotor area V6A (Galletti et al., 1999b). This latter, in turn, is subdivided in two cortical subsectors, a dorsal one named V6Ad and a ventral one named V6Av (Luppino et al., 2005; Gamberini et al., 2011).

### Retinotopic organization of macaque area V6

**Figure 2:**
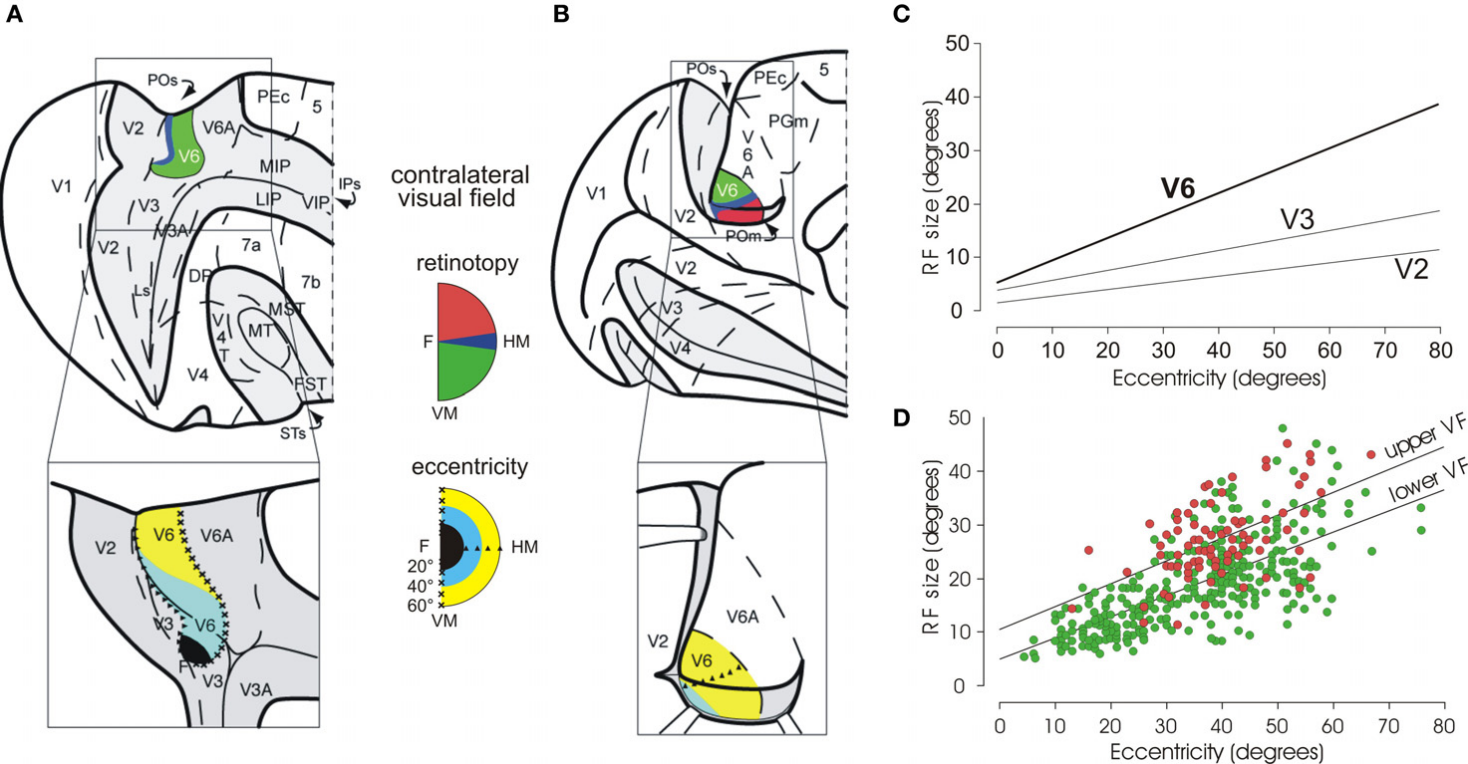
**Visual topography and receptive field properties of macaque area V6. (A)** Dorsal view of caudal half of right hemisphere (and, below, enlargement of the parieto-occipital region) with the parieto-occipital (POs), lunate (Ls), and intraparietal (IPs) sulci shown opened to reveal the cortex buried within them (dark gray area). **(B)** Medial view of the caudal half of a the left hemisphere (and, below, enlargement of the parieto-occipital region), with the medial parieto-occipital sulcus (POs) opened. Area V6 is shown in color, according to the part of visual field it represents (conventions reported between **A** and **B**). Note that V6 represents point-to-point the entire contralateral visual field, with an emphasis in the representation of the peripheral visual field. Triangles and crosses indicate the representation of the horizontal (HM) and vertical (VM) meridians of area V6, respectively; the F, the center of gaze. Dashed lines are the borders between different cortical areas. PEc, 5, MIP, LIP, VIP, 7a, 7b, MT, MST, V4, V4T, FST, PGm: areas functionally or anatomically identified in the posterior part of the cerebral hemisphere. Modified from Pitzalis et al. (2006). **(C)** Regression plots of receptive-field size (square root of area) against eccentricity for cells recorded in areas V2, V3, and V6. Receptive-field size increases with eccentricity in all visual areas. In area V6, receptive fields are larger than in V2 and V3 at any eccentricity. **(D)** Dual regression plot of RF size against eccentricity for RF in the upper (red circles) or lower (green circles) visual fields (VF), respectively. Modified from Galletti et al. (1999a). It is evident that at any eccentricity, RFs are bigger in the upper VF with respect to the lower one.

As shown in Figure 2, area V6 is topographically organized. The representation of the central part of the visual field, including the fovea, is located laterally in the POs, adjacent to area V3A, while the periphery is located medially, in the POs and the mesial surface of the hemisphere (Galletti et al., 1999a). V6 has a complete representation of the contralateral visual field with the lower quadrant represented in the POs and the upper quadrant in the mesial surface of the hemisphere. The vertical meridian is represented anteriorly, at the border with area V6A, and the horizontal meridian posteriorly, at the border with areas V2–V3. Both center and periphery of the visual field are uniformly represented, with a very low, if any, cortical magnification factor. Accordingly, a wider extent of cortex in V6 is devoted to the analysis of visual information in the periphery of the visual field.

Visual receptive fields (RF) in area V6 are larger than in areas V2 and V3. In V6, RF size increases with eccentricity (i.e., distance from the center of gaze), as in V2 and V3, but it remains on average larger than in V2 and V3 at any value of eccentricity (see Figure 2C). The size and distribution of RF in the upper and lower visual fields is unequal in V6: the RF located in the lower hemifield are smaller and more numerous with respect to those located in the upper visual field (Figure 2D). As a functional counterpart, the lower visual field could subserve a finer analysis of visual images with respect to the upper visual field, creating an advantage for processing visual information in the hemifield (the lower one) where we mostly move our arms and hands when interacting with external objects. This is in line with the fact that V6 is strictly connected with V6A (Galletti et al., 2001; Gamberini et al., 2009), a visuomotor parietal area intensively involved in the control of reach-to-grasp activity (Fattori et al., 2001, 2005, 2009, 2010; Marzocchi et al., 2008). It is likely that V6 provides V6A with specific visual information on the movements of arm/hand that are approaching to the object to be grasped.

### Visual motion properties of monkey V6

**Figure 3:**
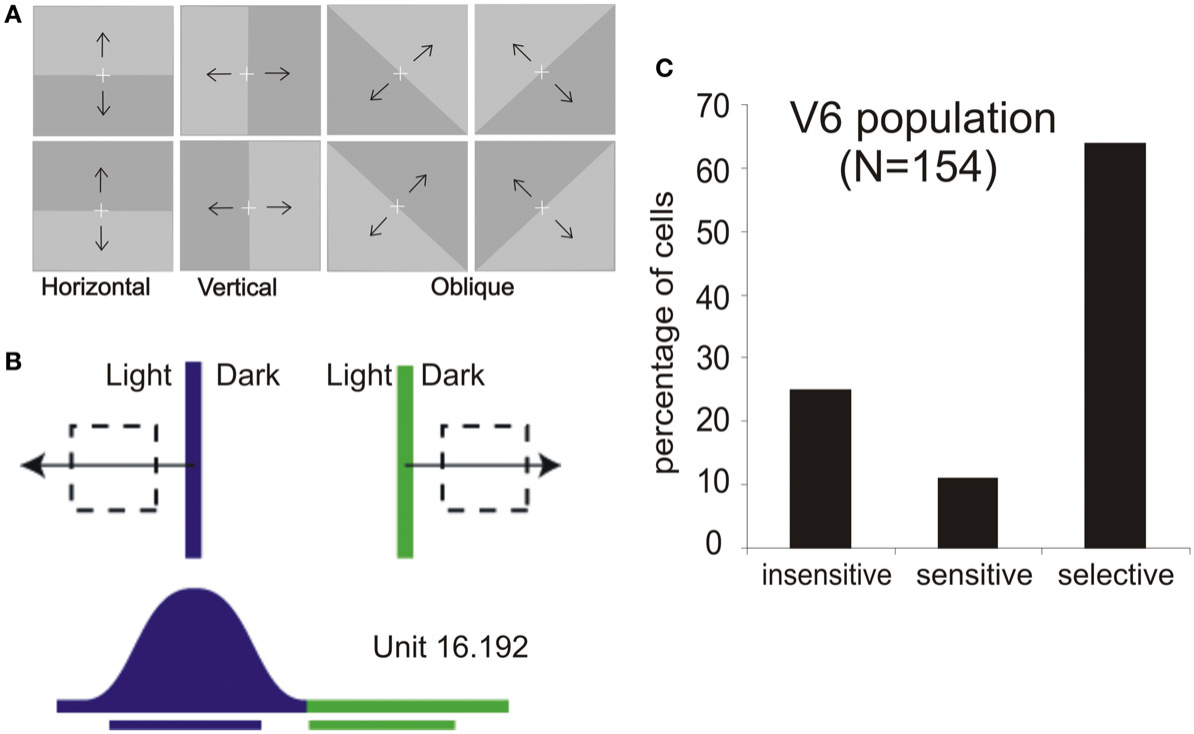
**Motion sensitivity in macaque area V6. (A)** Stimuli used for visual stimulation of V6. Light/Dark borders of different orientations and moving in different directions as indicated by the black arrows, vertically, horizontally, and in two oblique directions are the best stimuli to activate single cells in this cortical area. White cross: fixation point. **(B)** Visual response of a direction-selective V6 cell. Top: schematic representation of the receptive field (dashed line) and of the stimulus (light/dark border) moved across the receptive field in the direction indicated by the arrow (blue, leftward movement direction; green, rightward). Bottom: Scheme of the neural responses to the two directions of motion and bars indicating the durations of visual stimulations. **(C)** Incidence of direction sensitivity in V6 neuronal population. We defined as direction “selective” those cells whose response to a correctly oriented stimulus moving in the direction opposite to the preferred one was <20% of the firing rate evoked during optimal stimulation; direction “sensitive” those whose response was between 20% and 80%; and direction “insensitive” those whose response in the opposite direction was >80% of that in the preferred one. Modified from Pitzalis et al. (2010).

The stimuli more adequate to evoke brisk visual responses in V6 were light or dark bars, or single wide light/dark borders, moved across the receptive field (Figure 3A; Galletti et al., 1996, 1999a). The majority of V6 neurons are motion and direction sensitive: they respond to stimuli moving across their receptive field with certain velocities and having a specific direction of motion. Very often (in above 70% of cells), the same stimulus moving with the same velocity but in the opposite direction of movement does not evoke any discharge at all. An example of such kind of direction-selective responses is shown in Figure 3B and the relative incidence of direction-selective cells in the V6 population in Figure 3C.

Although the sensitivity of V6 cells to optical flow stimulations has not been tested to date, the presence of strong directional sensitivity in this area, together with the wide representation of the periphery, suggested us that V6 “could be engaged in the analysis of flow field resulting from self-motion” (see Galletti et al., 1999a). This hypothesis has now been tested in fMRI and VEP experiments in humans (Pitzalis et al., 2010, 2012b), as described in the following sections.

### Real-motion detection in V6

**Figure 4:**
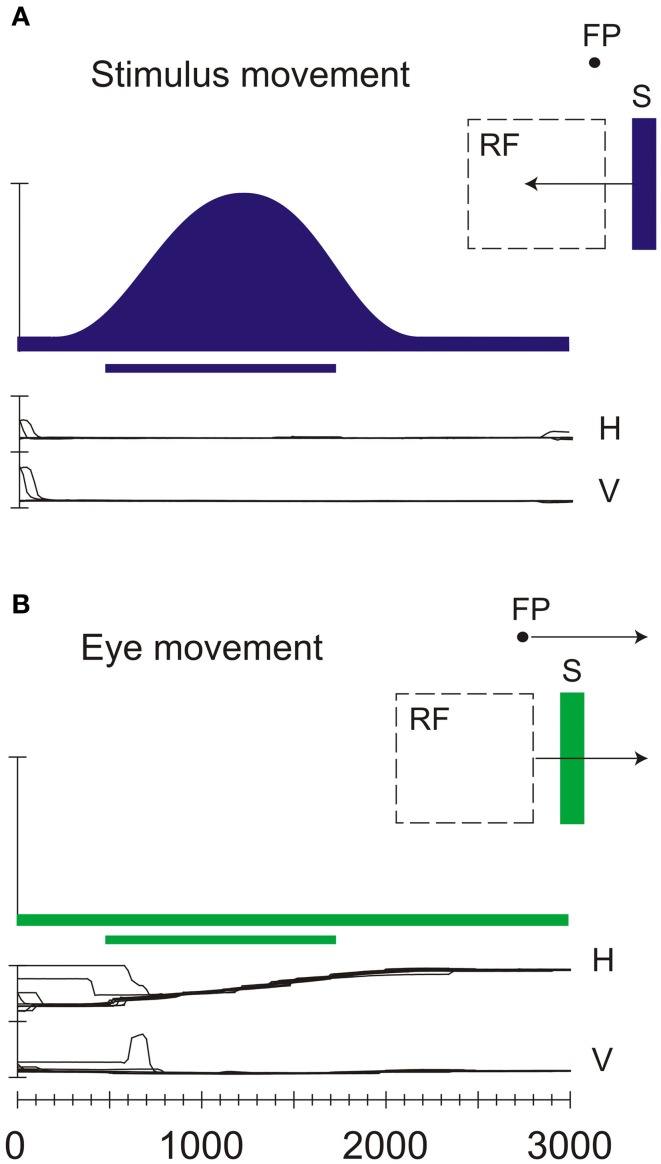
**Neural discharges of a real-motion cell recorded in area V6. (A)** Neural responses evoked by sweeping an optimal visual stimulus (S) across the receptive field (RF) while the animal looked at a stationary fixation point (FP). H and V indicate the horizontal and vertical components, respectively, of the eye movements. **(B)** Neural activity evoked by sweeping the receptive field across the stationary visual stimulus thanks to the pursuit eye movement evoked by the movement of the fixation spot. All conventions are as in Figure 2B. Modified from Galletti and Fattori (2003).

V6 is rich in a particular type of motion sensitive neurons, called “real motion cells” (Galletti and Fattori, 2003), that have been found, even in a smaller percentage, also in areas V1 (Galletti et al., 1984), V2 (Galletti et al., 1988), and V3A (Galletti et al., 1990). These real motion cells discharge vigorously for stimuli moving in a certain direction when the monkey is fixating on a point and the stimulus is moving in the neuron receptive field. However, when the same image of the stimulus moves in the same direction on the retina because the monkey's eyes move while the object is stationary, the response of the real motion cells is attenuated, or completely suppressed. Figure 4 shows an example of real-motion behavior: the visual stimulation and the motion stimulation are identical in the two situations, but in A there is a real movement of the stimulus, whereas in B the stimulus is motionless (the movement of the retinal image being self-evoked by the eye movements). In the first case (A), when the visual stimulus is actually moving in the external world, the cell discharges strongly. In the second case (B), when the stimulus is motionless in space, the cell does not change its baseline activity despite an identical retinal stimulation.

The behavior of real-motion cells indicates that these cells are processing additional information beyond the retinal ones. In particular, to recognize whether the retinal stimulation is due to a real movement or is self-evoked by the eye movements, these cells must take into account also the eye movements. We demonstrated that the movement of the eyes *per se* did not influence the activity of real-motion cells, but it strongly increases the cell's response to the retinal image movement (Galletti et al., 1984, 1988, 1990).

Since V6 is strongly connected with the visuomotor area V6A (Galletti et al., 2001; Gamberini et al., 2009; Passarelli et al., 2011), that contains many reaching-related neurons and neurons modulated by the covert attention (Fattori et al., 2005; Galletti et al., 2010; Gamberini et al., 2011), we hypothesized that the real-motion cells of this area could signal the actual object movements with the purpose to orient the animal's attention toward moving objects, and to reach and grasp the moving objects or to avoid them particularly in a crowded structured environment.

## Area V6 in the human brain

### Recognition of human area V6

**Figure 5:**
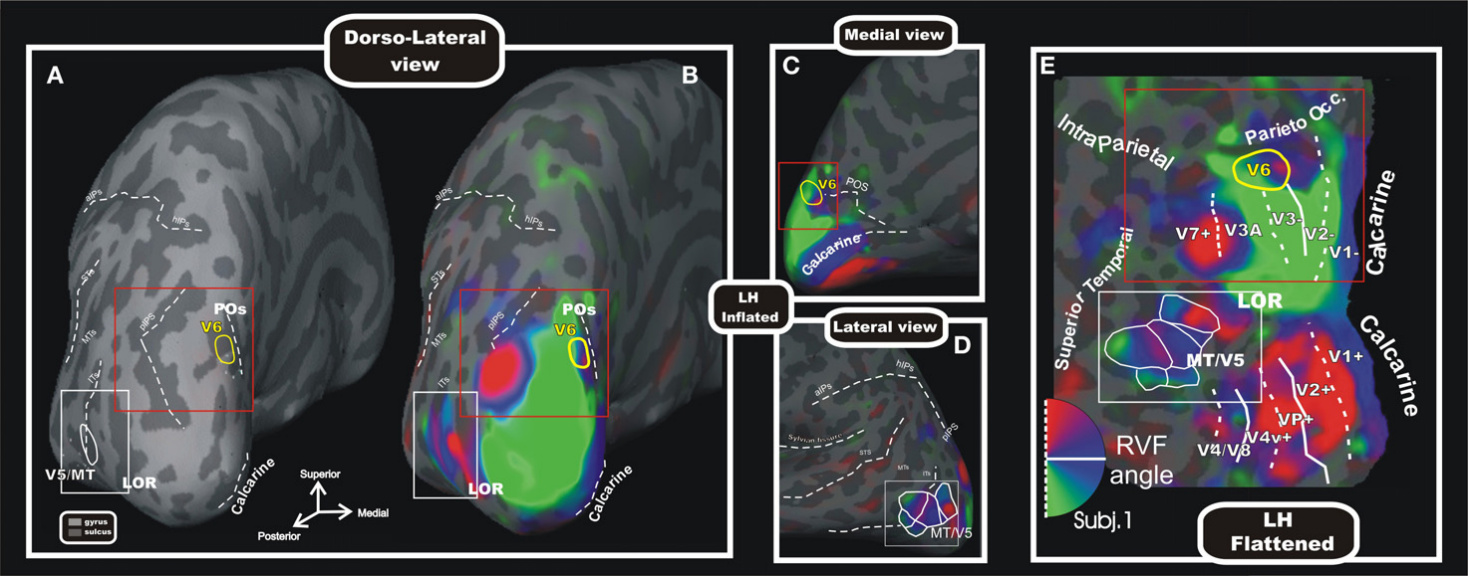
**Wide field retinotopy of polar angle representation of area V6 and MT/V5 in the human brain.** Inflated **(A–D)** and flattened **(E)** reconstructions of the left hemisphere (LH) of one participant are shown [modified from Pitzalis et al. (2006)]. Red and white boxes indicate trough the different views of the cortex the same parts of the brain where areas V6 and V5/MT are respectively located. Inflated cortex is shown in dorso-lateral view **(A,B)**, and on medial **(C)** and lateral **(D)** close-ups views of the posterior brain. **(A)** Reference image to show the typical position of areas V6 and MT/V5 respect to the medial sulci (POs, calcarine), the pIPS and the lateral middle temporal sulci (ITs, MTs, and STs). The cortical surfaces were defined at the gray-white matter border and have been inflated to reveal regions within the sulci (concavities, dark gray) as well as on the gyri (convexities, light gray). **(E)** Flattened map shows retinotopic phase-encoded signal in the dorsal and ventral cortical areas (including medial V6 and lateral MT/V5). The boundaries of all visual areas were defined by mapping visual field sign (Sereno et al., 1994, 1995). Dotted and solid white lines reported on the flat maps indicate vertical and horizontal meridians, respectively. In all sections, color hue indicates the response phase, which is proportional to the polar angle of the local visual field representation: green/blue/red areas represents lower/horizontal/upper fields, respectively (see hemifield icon in **E**). Yellow and white outlines indicate respectively location and borders of the human area V6 (Pitzalis et al., 2006) and MT/V5 (Pitzalis et al., 2010). Red and white boxes indicate through the different views of the cortex the same parts of the brain where areas V6 and V5/MT are respectively located. Major sulci (dark gray) are labeled as follows: POs, parieto-occipital sulcus; LOR, Lateral Occipital Region; pIPs, posterior end of the intraparietal sulcus; aIPs, ascending segment of the intraparietal sulcus; hIPs, horizontal segment of the intraparietal sulcus; STs, superior temporal sulcus; MTs, middle temporal sulcus; Its, inferior temporal sulcus. On the inflated surfaces, the fundi (dashed lines) of calcarine, sylvian fissure, aIPs, hIPs, pIPs, and POs are shown.

Since the macaque V6 was originally described as an extrastriate area that was retinotopically organized and that presented constant spatial relationship with the nearby retinotopically organized areas V2, V3, and V3A (Galletti et al., 1999a), the search of the human homolog of macaque area V6 was carried out by a retinotopic mapping. To map human V6 we used standard brain mapping methods, as the MRI for the cortical surface reconstruction and the fMRI in combination with the retinotopic mapping stimulations. However, given the great emphasis for the periphery of V6 in the macaque, we implemented an innovative set-up able to stimulate the entire visual field up to 110° in total visual extent, simulating for the first time in the fMRI scanner the conditions used in the study of monkey area V6 (Pitzalis et al., 2006). Wide-field retinotopic mapping (Figure 5) revealed that the retinotopic organization and neighbor relations of human V6 closely resemble those reported for macaque V6 (Galletti et al., 1999a; Pitzalis et al., 2006). Human V6, like macaque V6, is located in the POs and represents the entire contralateral hemifield, from the fovea to the far periphery (Pitzalis et al., 2006). V6 includes a medially located “upper” field representation distinct from the upper-field representation in lateral area V3A (Figures 5B,C,D,E). Human V6, like macaque V6, represents the fovea laterally (distinct from V3A), emphasizes the visual periphery and contains a mirror-image representation of the visual field (Pitzalis et al., 2006; Fattori et al., 2009).

The wide field retinotopy resulted in improved maps also in the lateral occipital cortex and MT+ (Figures 5B,C,D,E). In particular, the polar-angle maps confirmed the presence of an anterior facing upper-field representation in MT/V5, in line with previous reports (e.g., Huk et al., 2002) and with data from nonhuman primates (Allman and Kaas, 1971; Gattass and Gross, 1981). We also observed a number of other polar-angle maps around MT/V5 (Figure 5E), which resemble the mosaic of small areas found around nonhuman primate MT (Gattass and Gross, 1981; Van Essen et al., 1981; Desimone and Ungerleider, 1986; Sereno et al., 1994). The discovery of a mosaic of small retinotopic areas around retinotopic MT/V5 found in Pitzalis et al. (2010) was confirmed also in a recent fMRI study (Kolster et al., 2010) and fits with the hypothesis that the large motion-sensitive region MT is probably a complex of several areas, and for this reason it is typically labeled MT+.

### Human V6: a motion area highly sensitive to flow fields and translational egomotion

**Figure 6:**
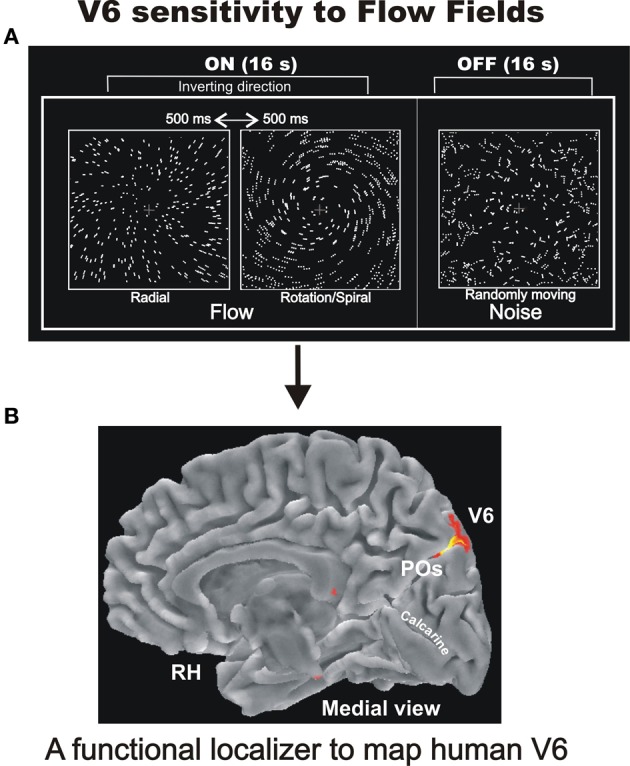
**Sensitivity of human V6 to Flow Fields. (A)** Flowfields stimulus. The two frames of the ON phase show the two different types of coherent motion (radial and rotation/spiral motion) that switched almost every 500 ms and were compared to random motion presented during the OFF phase. For both radial and spiral motion, we tested both expansion and contraction components [modified from Pitzalis et al. (2010)]. We used a wide field stimulation (up to 110°) and the subjects were instructed to fixate the central red cross to minimize eye movements. See methods in Pitzalis et al. (2010) for further details. **(B)** Area V6 mapped with the flowfields stimulus as a functional localizer. Results are displayed on the medial folded representation of the right hemisphere (RH) of the template brain. POs, parieto-occipital sulcus. Modified from Sdoia et al. (2009).

After having retinotopically defined the human area V6, several fMRI experiment have been performed to further investigate its functional organization with the more general aim to shed lights on its functional role within the dorsal stream. Several previous neuroimaging studies in humans showed that medial parieto-occipital cortex is activated by tasks involving visual motion perception (e.g., Cheng et al., 1995; Brandt et al., 1998; Galati et al., 1999; Sereno et al., 2001; Kleinschmidt et al., 2002; Kovács et al., 2008), but none of them, of course, directly related the activated region to the still unknown area V6. Thus, first we tested whether human V6 is motion sensitive like macaque V6, and if it was possible to identify an optimal visual stimulus for quickly localizing this area in fMRI studies, as it is typically done for MT+ (e.g., Tootell et al., 1995). Therefore, in a recent study of our group (Pitzalis et al., 2010), we first mapped the retinotopic organization of area V6 in single subjects as described in Pitzalis et al. (2006), and then we used several motion stimuli (including stimuli similar to those that were effective in activating cells in macaque area V6, see Figure 3A) as well as flickering stimulation (like that previously used in activating the medial parieto-occipital cortex; Portin and Hari, 1999; Portin et al., 1999; Dechent and Frahm, 2003) to see if they activate V6 (Pitzalis et al., 2010). Our results revealed that human V6, like macaque V6, is a motion area that responds to unidirectional motion (Drifting Edges). Human V6 was also sensitive to coherent Flow Field motion (Figure 6A) and to flickering stimulation.

The Flow Fields stimulus (not previously tested in macaque V6 by single unit recordings) was in fact the most effective visual stimulus in driving human V6 in fMRI experiments, both at individual and group levels and even with visual stimuli of standard size. Hence, we suggested it as a good functional localizer for the area (Figure 6B; Pitzalis et al., 2010). It is worthwhile to note that the functional localizer is an easier and faster tool to map V6 than the demanding retinotopic mapping, does not need the use of a wide field stimulation, takes only 4 min at shot, and is generally applicable in any fMRI lab.

The Flow Fields stimulus used in Pitzalis et al. (2010; Figure 6B) was a type of complex coherent motion stimulation similar to the continuously changing optic flow generated when a person moves through in a complex environment (Koenderink, 1986). Optic flow is probably the most important visual cue for perception of self-motion or -also called- “egomotion” (i.e., the sensation to be moving in space). The strong activation of V6 we observed in the wide-field Flow Fields experiments (that were very powerful in inducing a compelling perception of vection, i.e., illusory egomotion) suggested that V6 could be involved in the analysis of egomotion. This hypothesis could also be advanced on the basis of several previous functional imaging studies reporting activation in the medial parieto-occipital cortex for coherent wide-field stimuli, such as patterns simulating forward self-motion (e.g., Cheng et al., 1995; Brandt et al., 1998; Galati et al., 1999; Previc et al., 2000; Kleinschmidt et al., 2002; Kovács et al., 2008), but none of them, again, directly related the activated region to the still unknown motion area V6. Yet in agreement with this view, human clinical studies reported that lesions or electrical stimulation of the cortex of human POs produce motion-related visual disturbance (e.g., Heide et al., 1990; Richer et al., 1991; Blanke et al., 2003), and epileptic seizures within the precuneus produce linear self-motion perception (Wiest et al., 2004).

Egomotion can be experienced along different planes and cardinal axes depending on the *type* of self-movement (Gibson, 1966; Koenderink, 1986; Morrone et al., 2000). The optic flow that is generated when a person moves through the environment can be locally decomposed into several basic components, including radial, circular, translational, and spiral motion (see Hixson et al., 1966 for planes and cardinal axes nomenclature). Despite several neuroimaging studies have investigated the neural bases of egomotion (e.g., Tootell et al., 1995; Morrone et al., 2000; Kleinschmidt et al., 2002; Wall and Smith, 2008; Cardin and Smith, 2010; Pitzalis et al., 2010) the specific role of the different cortical regions in distinguishing different visual egomotion signals has not yet been determined because their peculiar sensitivity to different types of egomotion-compatible optical flows has never been tested.

**Figure 7:**
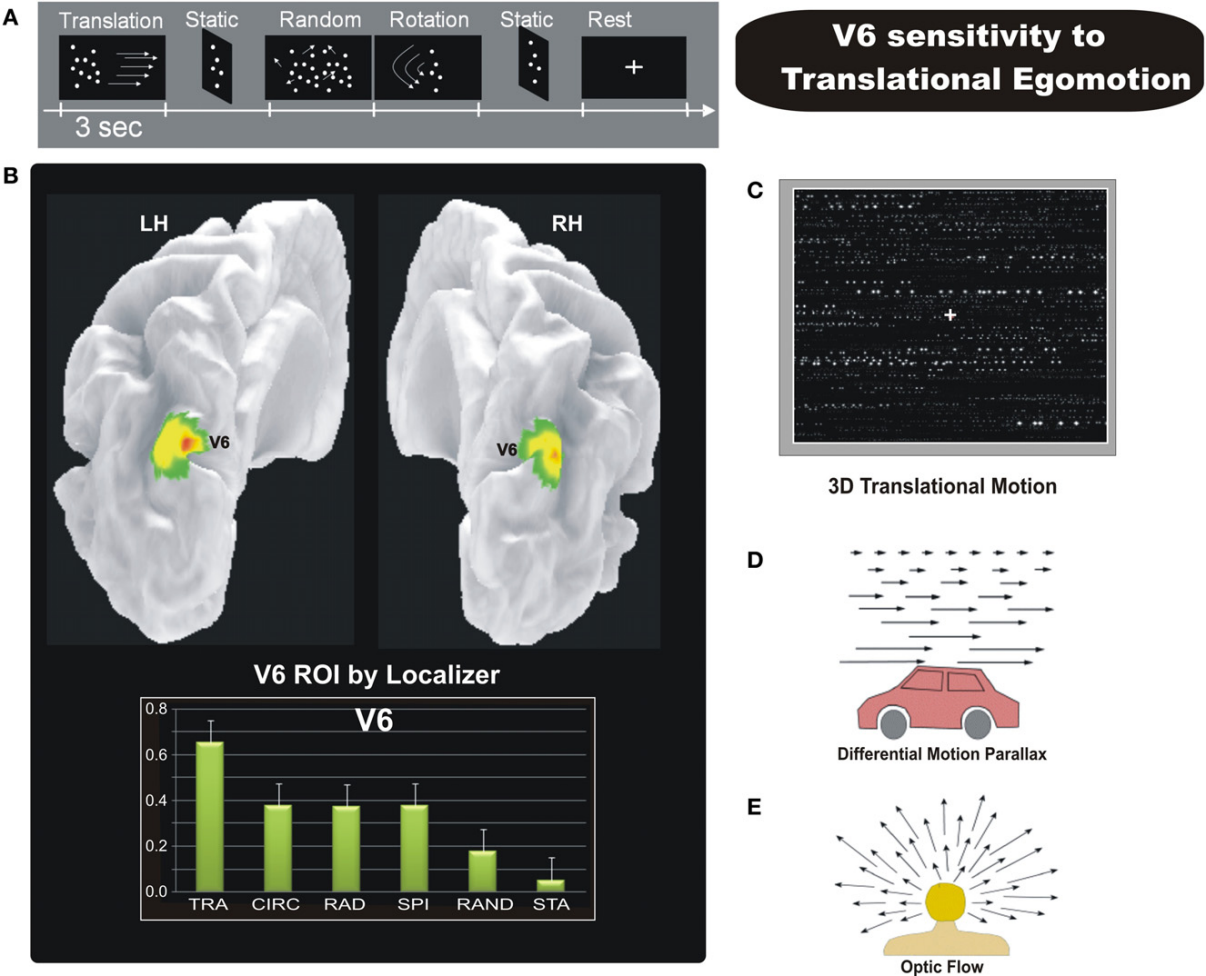
**Sensitivity of human V6 to Translational egomotion. (A)** Schematic representation of the visual stimulation sequence used in the event-related fMRI experiment. **(B)** *Top:* Area V6 mapped with the functional localizer, i.e., coherent flow vs. randomly moving dots (shown in Figure 6A). Results are displayed on a posterior dorso-medial view of the medial folded representation of the left and right hemisphere of the template brain. *Bottom*: The plots represent the averaged BOLD percent signal change across subjects and hemispheres in the localizer-defined area V6 for each experimental condition labelled as follows: TRA, translational; CIR, circular; RAD, radial; SPI, spiral; RAND, random; STA, static. **(C)** Sketch of the tridimensional translation stimulus used in the fMRI experiment. [**A–C** Modified from Sdoia et al. (2009)]. **(D)** Differential motion parallax. Differential motion parallax is the perceived difference in speed of nearby objects compared to far away ones. Motion parallax is easily perceived when we look through a car window, the velocity of nearby objects appears to be greater than that of distant objects. **(E)** Optic flow occurs when we are moving in a particular direction. If we look toward the point to which we are heading (the focus of expansion) this doesn't show movement, whereas by looking at the surrounding space, the visual field appears to be expanding. This effect can be perceived with great accuracy by the human brain, contributing to the control of locomotion and helping to continue heading toward the specified location. This effect gives the sense of movement that occurs when we see the famous star field screensaver, which produces the effect of navigating through a field of stars, heading toward a particular point on the screen. [**D–E** Modified from Ware (1999) “Information Visualization”].

We therefore performed an event-related fMRI experiment (Figure 7A) to explore the sensitivity to different types of egomotion-compatible visual stimulations in area V6, and in other human motion-sensitive regions such as areas MT, MST, V3A, CSv (cingulate sulcus visual area), and VIP (Ventral Intraparietal) (Sdoia et al., 2009). As visual stimuli, we used “*star fields*” designed to add the depth to the visual stimulation. With a wide field stimulation, the subject felt to be immersed in the flow patterns and experienced vivid sensations of different types of egomotion as those experienced in a 3D environment. Star fields simulated various flow patterns consistent with different movement of the observer, as radial (moving observer forward or backward along the line of sight), translational (translating observer horizontally, Figure 7C), circular (rotating observer around the line of sight) and spiral (forward or backward rotating observer with an added rotational component). Importantly, the behavioral results of preliminary psychophysical experiments showed that the four coherent motion conditions we used were all able to evoke a strong illusory egomotion sensation, each along a different plane. As shown in Figure 7B, fMRI results revealed a strong preference of V6 for coherent motion, confirming previous fMRI studies (von Pföstl et al., 2009; Cardin and Smith, 2010; Pitzalis et al., 2010; Helfrich et al., 2012). We found that only three cortical motion areas (V6, VIP, and MST) are able to distinguish among different types of self-movements. All the three areas showed a high response for translational egomotion, maximally in V6 and VIP and less marked in MST. In contrast, areas MT and V3A were not affected by the various types of optic flow. The visual area CSv, which has recently been shown to be activated by both visual self-motion information (Cardin and Smith, 2010; Fischer et al., 2012) and vestibular stimulations (Smith et al., 2012), surprisingly was weakly activated by coherent motion but robustly inhibited by random motion and static stimuli.

Overall, results shown in Sdoia et al. (2009) confirmed that human area V6 is suitable for the analysis of egomotion, as initially suggested in Pitzalis et al. (2010), and additionally showed that V6 is able to distinguish between different 3D flow fields, which is a necessary prerequisite for an area processing egomotion signals (e.g., Duffy, 1998). The view that V6 is involved in the estimation of egomotion has been tested also in other recent fMRI studies. Indeed, in humans this area has been shown to be sensitive to optic flow in particular if it is compatible with self-motion (Cardin and Smith, 2010). Furthermore, Cardin and Smith (2011) have shown that in V6, sensitivity to optic flow patterns is enhanced when they are combined with binocular disparity cues that are consistent with self-motion. An area is considered more well-suited to self-movement perception if it is also influenced by vestibular signals. Recently Smith et al. (2012) found that, MST is activated by vestibular stimuli, while V6 and VIP surprisingly do not appear to have vestibular input. They used a Galvanic Vestibular Stimulation (GVS). As also stated by the same authors, a lack of activity during GVS does not necessarily indicate that a particular region is unaffected by vestibular stimuli. Translational egomotion sensitivity is associated to otolithic activity while the rotational perceptual response reflects-induced neural activity evoked by stimulation of the semicircular canals. Cortical regions that are concerned mainly with translational egomotion could be little affected by GVS despite receiving otolithic signals, and this could be the case for areas V6 and VIP. Overall, all the studies reviewed above suggest that human area V6 is suitable for the analysis of egomotion.

The selective preference of V6, VIP, and, partly, MST for the 3D translational egomotion (Figure 7C) shown in Sdoia et al. (2009) raises the question of its functional significance. The translational motion condition used here simulate an observer translating horizontally, such as for example, when we are on a moving train or car while looking on the lateral window (Figure 7D). In physiologic conditions, during body translation in the horizontal plane the retinal motion of objects located at different distances respect to the observer generates the *differential motion parallax*, that is the perceived difference in *speed* and *direction* of nearby objects compared to far away ones. This powerful depth cue enables us to evaluate the relative distance of near and far objects in the environment. It is worthy to note that translational flow is thus conceptually different with respect to the other flow patterns. The spiral and radial 3D flow stimuli used here, for instance, produced the effect of navigating through a field of stars, heading toward a particular point on the screen (the focus of expansion) (Figure 7E). In the translational optic flow, in contrast, the accent is not on the heading direction but on the lateral visual flow produced by near and far external objects. Therefore the translational stimulus gives the possibility to evaluate the depth of objects in a dynamic condition such as that created by self-motion. The strong response to translational motion observed particularly in areas V6 and VIP suggests that these areas process visual egomotion signals to extract information about the relative distance of objects, likely in order to act on them, or to avoid them.

Interestingly, despite the V6 preference for self-motion over other types of global motion, a recent study surprisingly suggests that V6 does not encode direction of heading (Cardin et al., 2012). Given that disparity is most informative for nearby objects that generate relatively large retinal disparities, the authors suggested that V6 may be concerned with flow for the purpose of avoiding obstacles during self-motion rather than for providing a representation of heading direction (Cardin et al., 2012). The selective preference of V6 for the 3D translational egomotion shown in Sdoia et al. (2009) lends support to this view.

**Figure 8:**
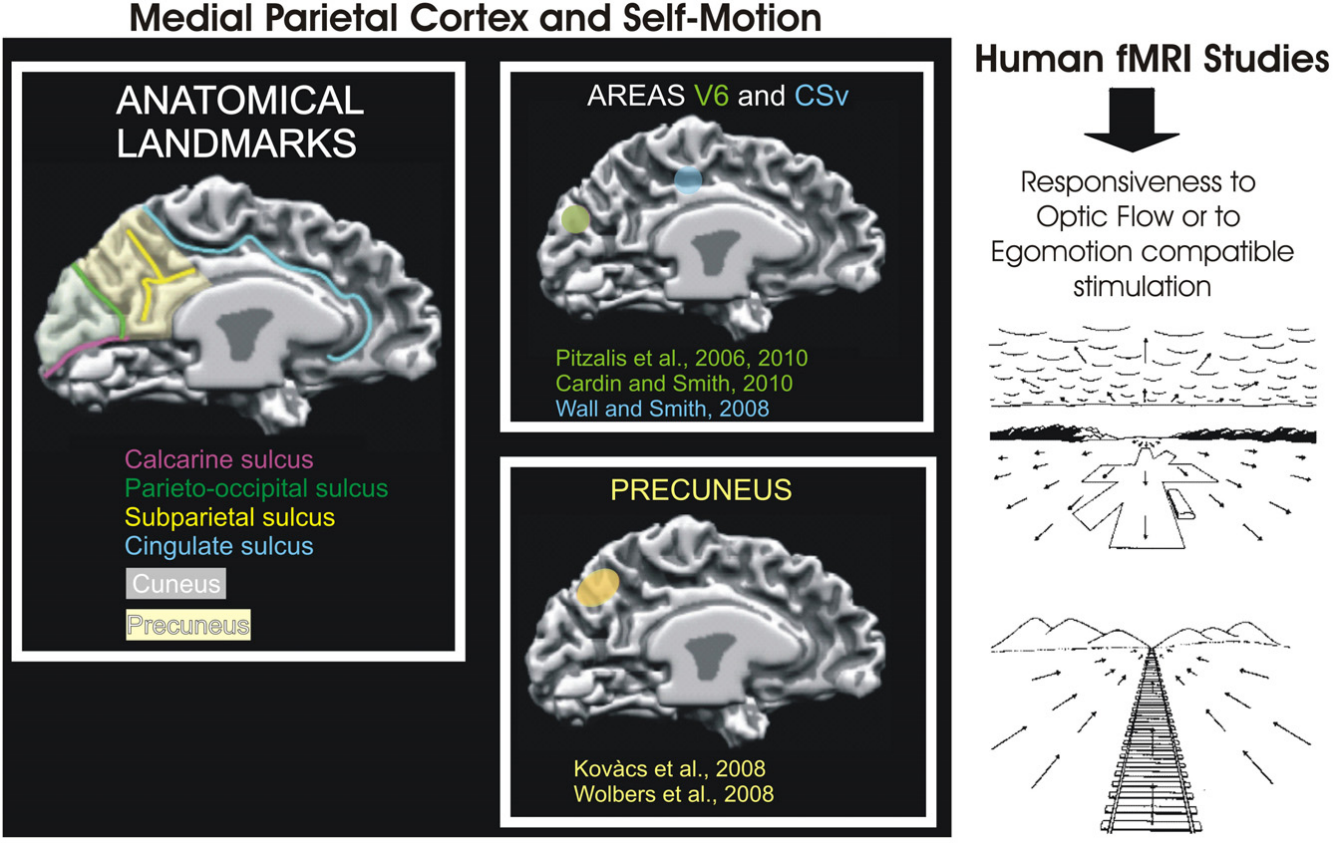
**Medial Parietal Cortex and Self-Motion. Left**: Schematic representation of motion-related areas within the human superior parieto-occipital cortex in some fMRI studies. Activation foci are shown on the medial surface of one representative subject's left hemisphere. The cortical surface was defined at the gray-white matter border and has been partially inflated to reveal regions within the sulci (concavities, in dark gray) and on the gyri (convexities, in light gray). Foci are schematically represented based on their sizes and anatomical locations relative to the parieto-occipital, calcarine, and cingulate sulci, as depicted in figures from the original studies: V6 (green), retinotopy (Pitzalis et al., 2006) and function (Cardin and Smith, 2010; Pitzalis et al., 2010); CSv (light blu) (Wall and Smith, 2008) and Precuneus (yellow) (Kovács et al., 2008; Wolbers et al., 2008). Modified from Culham et al., 2008. **Right**: Examples of retinal optic flow. Expanding optic flow during forward motion. Panel shows a typical retinal optic flow during a landing. The landing field, the mountains and the clouds are visible. Contracting optic flow during inward motion. Panel shows a typical retinal optic flow seen by an observer sitting on a moving train and looking in the opposite direction respect to the motion direction of the train. In both panels, the arrows indicate the optic flow direction. The arrows length is proportional to the speed of motion. Modified from Bruce et al. (1996).

Overall, since the definition of area V6 as a retinotopically organized motion area of the dorsal visual stream, many fMRI studies started to map it in their experiments (retinotopically or functionally) describing their results specifically referring to area V6 and not more to a generic activation of the medial parietal cortex as in the past. This important change had relevant implications in that it helped not only to delineate a more clear picture of the functional role of area V6, but also to increase the attention for the motion processing in the medial parietal cortex (Figure 8, see also Kravitz et al., 2011 for review).

### Human V6: an early station coding motion coherency

The functional role of a brain area can also be studied by analyzing its response timing, which can also provide important cues with respect to the pattern of its anatomical connections. The response timing of V6 and MT+ was studied using a combined VEPs/fMRI technique developed and utilized by our group in many previous studies (e.g., Di Russo et al., 2002, 2003, 2005, 2007, 2011; Pitzalis et al., 2012a). The increased resolution of combined EEG/fMRI methods enabled us to follow the flow of motion signals from the occipital pole to the medial and lateral motion areas V6 and MT+, and made it possible to localize the VEP data within each retinotopic visual area identified in individual subjects (e.g., Sereno et al., 1995; Pitzalis et al., 2006).

**Figure 9:**
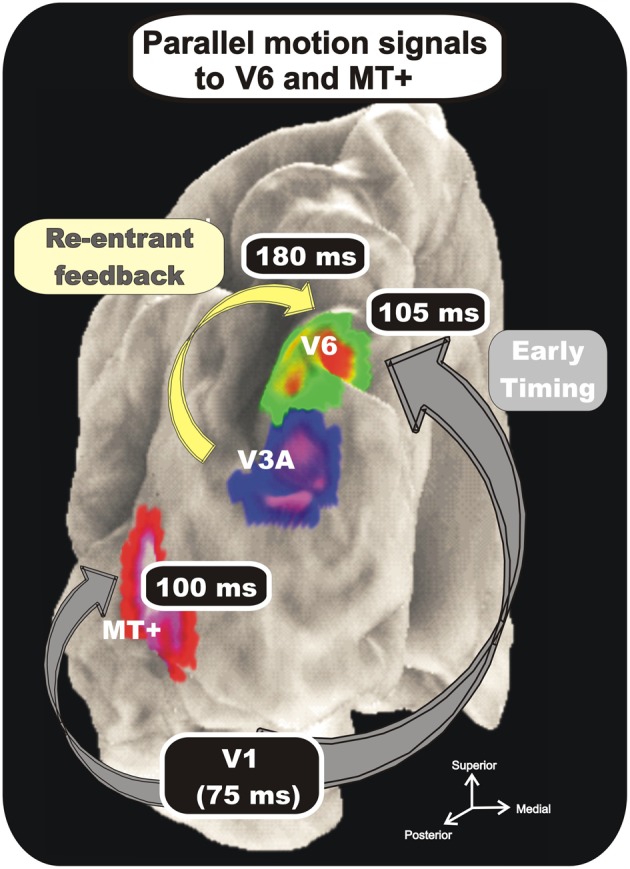
**Combination of VEP and fMRI data.** Group-averaged Imaging results. Regions more activated in the coherent or incoherent motion conditions (contrasts C-I and I-C) are indicated on the cortical surface using text labels (V6, V3A, and MT+) together with labels about their response timing. Results are displayed together on the semi-inflated cortical surface reconstruction of the left hemisphere of the average brain. Numbers indicate time of occurrence of VEP signals. Modified from Pitzalis et al. (2012b).

As expected, we found a strong preference of V6 for coherent motion which is in line with previous fMRI studies (von Pföstl et al., 2009; Sdoia et al., 2009; Cardin and Smith, 2010; Pitzalis et al., 2010; Helfrich et al., 2012). Additionally, we found that area V6 is one of the most early stations coding the motion coherence and that its electroencephalographic activity is almost simultaneous with that of MT+ (Figure 9). The early timing of V6 activation (onset latency 105 ms) together with the small temporal gap with the V1 timing (peak latency 75 ms) is in agreement with data on macaque brain, where it has been proved the existence of a direct connection between V1 and V6 (Galletti et al., 2001). This result fits also with previous human MEG studies which found visual activity in POs and V1 in a similar latency range- between 60 ms and 100 ms from stimulus onset (Vanni et al., 2001; von Pföstl et al., 2009). We also found a second, late peak of activity in V6 in the latency range of the P2. The same peak of activity was found in previous studies (Hoffmann and Bach, 2002; Kremlácek et al., 2004; Pitzalis et al., 2010; Di Russo et al., 2011) and was attributed to processing of complex features of motion (expanding/contracting radial motion) (see Kuba et al., 2007 for review). In Pitzalis et al. (2012b) we showed that the analysis of such complex motion signals also occurs much earlier, about 100 ms before (N140), supporting the hypothesis of a V6 involvement in early cortical motion processing. We interpreted the late activity in V6 (P230) as a re-entrant feedback from other extrastriate visual areas, like V3A which in the macaque is strongly connected with V6 (Galletti et al., 2001). V3A activity is supposed to be involved in the analysis of motion, since it contains many real-motion cells that are able to distinguish between real object motion and motion of retinal images that are self-induced by eye movements (Galletti et al., 1990), and in extracting form from motion (Zeki, 1978; Vanduffel et al., 2002). Such a type of signal could help V6 to recognize real motion of objects among the plethora of retinal image movements self-evoked by eye and body movements (Galletti and Fattori, 2003).

In summary, the analysis of our VEPs/fMRI data show a rapid sequence of activation from the occipital pole to areas V6 and MT+. These two dorsal motion areas have similar response onset latencies (100 ms and 105 ms), with a delay of about 25 ms with respect to V1 peak. The minimal temporal gap between the two areas supports the view of direct interconnections between V1 and the two motion areas, as found in the macaque brain (Shipp and Zeki, 1989; Galletti et al., 2001). It could also be that V6 and MT+ are anatomically interconnected, as it is the case in the macaque monkey (Galletti et al., 2001). Previous MEG studies, although not recognizing specifically areas V6 and V5/MT, can be considered in agreement with this view. Vanni and coworkers (Vanni et al., 2001; von Pföstl et al., 2009) reported in fact that visual activity in dorsal POs (putatively area V6) and in V1 have a similar latency range, and Tzelepi et al. (2001) reported no significant differences between the onset latencies of the POs and the temporal occipital (TO) region, which likely includes the motion sensitive MT+.

## Two motion areas in the dorsal visual stream

We have demonstrated the existence of a new *medial* motion-sensitive area (V6) distinct from the classic *lateral* motion-sensitive area MT+ in the dorsal visual stream of both human and non-human primates (Galletti et al., 1996, 1999a; Pitzalis et al., 2006, 2010). The two motion areas, though located in quite separate parts of the brain, receive a direct input from the striate cortex and share several functional properties relative to the analysis of motion, as direction selectivity, and speed preference (e.g., Galletti and Fattori, 2003).

According to revised versions of the anatomo-functional organization of the primate dorsal visual stream (see Rizzolatti and Matelli, 2003; Galletti et al., 2003 for review) there would be two distinct functional systems: a dorsomedial occipito-parietal pathway passing through V6 and V6A (Galletti et al., 2001, 2003; Gamberini et al., 2009) and a dorsolateral occipito-temporal-parietal pathway passing through MT/MST (see Galletti et al., 2001 for these data in the monkey brain). Both streams are responsible for action organization, however, while the major functional role of the dorsomedial occipito-parietal stream is the control of actions “on line,” the dorsolateral occipito-temporal-parietal stream plays a crucial role in space perception and action understanding. Thus, although MT/V5 and V6 are strictly interconnected one another and are both involved in the analysis of motion in the visual field, they should be considered two-independent motion processors having partly different output: (while MT/V5 projects mostly to areas of the inferior parietal lobule, area V6 is mainly connected with the SPL) and likely different functions.

On the functional point of view, we found a functional dissociation between V6 and MT+ (Pitzalis et al., 2010) and a general different functional profile when different coherent and incoherent motion stimuli are used (Pitzalis et al., 2012b). We found also that unlike V6, MT is not able to distinguish between different 3D flow fields (Sdoia et al., 2009). These data suggest that the two dorsal motion areas play different functional roles, a view that emerged also from other laboratories in the last decade (e.g., Kravitz et al., 2011). We have suggested some years ago that V5/MT is involved in the analysis of motion signals (direction and speed of movement), particularly in the central part of the visual field, whereas V6 in both object and self-motion recognition across the whole visual field (see Galletti and Fattori, 2003). The small temporal gap between the onset of visual responses in areas MT+ and V6 (Pitzalis et al., 2012b) and the strong interconnection between the two areas (Galletti and Fattori, 2003) lend support to this view. All the recent neuroimaging results from ours and other laboratories reviewed before demonstrated that human V6 is a medial motion area involved in estimation of egomotion (Sdoia et al., 2009; Cardin and Smith, 2010, 2011; Pitzalis et al., 2010, 2012b; Fischer et al., 2012). Recent fMRI data suggest that also human V6 contains real-motion cells, since the activity of a medial posterior parietal region that likely includes V6 correlates predominantly with the real motion of objects in the visual field (e.g., Tikhonov et al., 2004; Bartels et al., 2008). Additionally, area V6 has a strong response to translational egomotion (Sdoia et al., 2009) that suggests that this area processes visual egomotion signals to extract information about the relative distance of objects, likely in order to act on them, or to avoid them rather than for providing a representation of heading direction (see also Cardin et al., 2012). The hypothesis that V6 is involved in processing of motion of graspable objects is based also on its tight connectivity with areas involved in grasping (Galletti et al., 2001, 2003), its sensitivity to optic flow patterns combined with disparity cues, most informative for nearby objects (Cardin and Smith, 2011) and its putative preference to near-field stimuli in humans (Quinlan and Culham, 2007). Given this emphasis on objects, a possibility is that V6 is involved in “subtracting out” self-motion signals across the whole visual field for the purpose of flow parsing—the separation of object motion from self-motion (Warren and Rushton, 2009)- as suggested by our group (Galletti and Fattori, 2003; Pitzalis et al., 2010) as well as by other authors (Cardin et al., 2012). The segregation of these two types of motion is essential both for the avoidance of obstacles and for planning the handling of nearby objects, and it has been shown to use optic flow as well as local motion signals (Warren and Rushton, 2009).

**Figure 10:**
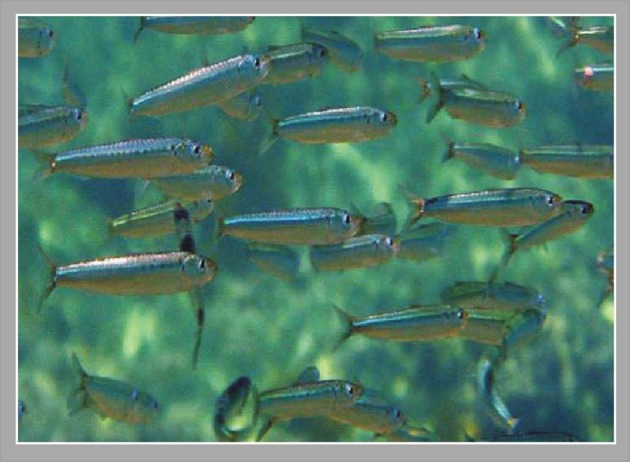
**Example of 3D translational motion in the natural environment.** A school of fish translating horizontally respect to the observer who is advancing under water toward the fishes.

From V6, visual information would reach bimodal visual/somatosensory areas in the superior parietal lobule (areas V6A and MIP) that are able to encode visual space and arm reaching movement (Colby and Duhamel, 1991; Galletti et al., 2001, 2003; Galletti and Fattori, 2002). Even though area V6 is not directly involved in the control of movement, its output is known to converge through area V6A on the dorsal premotor cortices (Shipp et al., 1998; Galletti et al., 2004; Gamberini et al., 2009). In turn, the premotor cortex controls the direction of arm movements toward objects in the peripersonal space. Thus, the ability of V6 cells to recognize the “real movement” in the visual field and to encode the direction of movement of objects could be useful to encode the continuously changing spatial location of moving objects, providing the spatial coordinates of moving targets to the controllers of arm reaching movements. We can suppose that information on objects in depth which are translating in space because of the self-motion are processed in V6 and conveyed to V6A for evaluating object distance in a dynamic condition such as that created by self-motion, so to orchestrate the eye and arm movements necessary to reach or avoid static and moving objects in the environment (e.g., swimming under water crossing a school of fish, Figure 10).

In summary, macaque and human results together suggest that V6 is a classical extrastriate visual area entirely devoted to the encoding of both object and self-motion, likely with the purpose of flow parsing. Given its high motion sensitivity, medial area V6 must be now considered another key motion region of the dorsal visual stream in both macaque and human brain as lateral area MT+.

### Conflict of interest statement

The authors declare that the research was conducted in the absence of any commercial or financial relationships that could be construed as a potential conflict of interest.
